# The invasive forest pathogen *Hymenoscyphus fraxineus* boosts mortality and triggers niche replacement of European ash (*Fraxinus excelsior*)

**DOI:** 10.1038/s41598-020-61990-4

**Published:** 2020-03-24

**Authors:** Olalla Díaz-Yáñez, Blas Mola-Yudego, Volkmar Timmermann, Mari Mette Tollefsrud, Ari M. Hietala, Jonàs Oliva

**Affiliations:** 1 School of Forest Sciences, University of Eastern Finland, PO Box 111, 80101 Joensuu, Finland; 2 NIBIO Norwegian Institute of Bioeconomy, P.Box 115, 1431 Ås, Norway; 3 NIBIO Norwegian Institute of Bioeconomy, P.Box 2609, 7734 Steinkjer, Norway; 4 Department Crop and Forest Sciences, University of Lleida, Lleida, Spain; 5 Joint Research Unit Agrotecnio - CTFC, Lleida, Spain; 6 Department Forest Mycology and Plant Pathology, Swedish University of Agricultural Sciences, Uppsala, Sweden

**Keywords:** Plant sciences, Forest ecology

## Abstract

Determining the impacts of invasive pathogens on tree mortality and growth is a difficult task, in particular in the case of species occurring naturally at low frequencies in mixed stands. In this study, we quantify such effects by comparing national forest inventory data collected before and after pathogen invasion. In Norway, *Fraxinus excelsior* is a minor species representing less than 1% of the trees in the forests and being attacked by the invasive pathogen *Hymenoscyphus fraxineus* since 2006. By studying deviations between inventories, we estimated a 74% higher-than-expected average ash mortality and a 13% slower-than-expected growth of the surviving ash trees, indicating a lack of compensation by the remaining ash. We could confidently assign mortality and growth losses to ash dieback as no mortality or growth shifts were observed for co-occurring tree species in the same plots. The mortality comparisons also show regional patterns with higher mortality in areas with the longest disease history in Norway. Considering that ash is currently mostly growing in mixed forests and that no signs of compensation were observed by the surviving ash trees, a significant habitat loss and niche replacement could be anticipated in the mid-term.

## Introduction

Global warming and globalization put pressure on forests ecosystems and threaten their capacity to deliver goods and services to society^1,2^. In the last centuries, an increasing number of invasive pathogens have been introduced into forests^3^ mainly by trade of living plants, which has created an unprecedented movement of species across continents^4–6^. All invasive species are not equally harming, and while some can have small, undetectable or even positive impacts^7^, others can cause high ecological and economic damage^8,9^. Invasive pathogens are recognized by the general public as a threat across Europe^10^, but evidence of their impact is needed in order to enforce an effective response from society^11,12^.

Invasive species can have impacts at different scales and with different intensities. There are some paradigmatic examples of fungal invaders that have caused large losses across vast areas in a short time, such as the chestnut blight killing American chestnuts, or the Dutch elm disease affecting European elms^7^. However, the impact of other invaders can be restricted to certain environments such as river banks or city parks^13^, or they cause a more diffuse damage, for instance by not killing but weakening trees and making them more susceptible to other biotic or abiotic stressors^1^. Such interactions can affect the vigour of the species and result in compositional changes in the long term.

One way to assess the impact of new pathogens is to look at the changes that they have caused to the population of the host. Manion and Griffin^14^ established a system to measure the impact posed by new pathogens by comparing tree mortality before and after pathogen arrival. Their model assumed that tree species growing in a forest have co-evolved with native pathogens, which target individuals weakened by inter- and intra-specific competition for resources. Changes in the population of the host would occur if the new pathogen caused a higher mortality than that occurring under natural conditions in the forest. Manion and Griffin^14^ obtained the baseline mortality from a *Liocourt* curve calculated from tree inventories carried out over large areas. This model shows how the density of trees decreases as they increase in diameter. The underlying process behind this curve is that weaker trees eventually die, and the surviving trees increase their size as they occupy the newly freed space. The model is adjusted so that the same decay or mortality operates regardless of tree size, and thus can be used as a ‘baseline mortality’ value to be compared with when analysing areas affected by a new pathogen. Manion and Griffin^14^ applied this approach to widespread tree species in eastern US; however, this model can be difficult to apply in the case of minor species, such as *Alnus glutinosa*, *Fraxinus excelsior* or *Ulmus minor*, affected by exotic pathogens in Europe. These species are poorly represented in national forest inventories (NFIs), and often appear in mixed natural stands where baseline mortality may be difficult to estimate.

In this study, we focus on European ash (*Fraxinus excelsior*) in Norway. Ash, a keystone species with wide distribution and habitat range in Europe, is being affected by the non-native fungus *Hymenoscyphus fraxineus* (T. Kowalski) Baral, Queloz & Hosoya. European ash grows over a wide range of altitudes and soil moisture contents, depending on climatic and site conditions. It is frequently mixed with other broadleaved trees from several genera, including *Acer*, *Alnus*, *Betula*, *Carpinus*, *Corylus*, *Populus*, *Salix*, *Ulmus* and *Quercus*^15,16^. Ash trees show a high regeneration capacity both in woodland and in non-woodland situations^17^, to the extent that the species has been considered invasive^15,18^. Ash dieback was first reported in Poland at the beginning of the 1990s^19^ and has since spread to most areas in Europe^12,20–22^, being first detected in Norway in 2008^23^. Based on the age of stem bark lesions, it was later concluded that the fungus had arrived to the region no later than 2006^24^. While the disease was initially identified in the south eastern Norway, since then it has been spreading northward along the west coast, advancing about 51 km annually^24^. The fungus causes cankers and kills shoots, which result in a progressive loss of functional crown, ultimately leading to tree death. Ash dieback affects ash trees of all ages, although younger trees are usually killed faster than intermediate or older ones. Dominant trees may stay alive for a number of years despite having a high degree of crown defoliation. In big trees, the crown condition may fluctuate to some extent between seasons as lesion extension or pathogen sporulation may vary according to seasonal weather conditions^25^.

We present a novel approach that assesses pathogen invasion related changes in mortality and growth trends of minor tree species by using national forest inventory data collected before and after the appearance of a new pathogen. In a first step, we model the mortality and growth of individual trees based on their size and the size of the rest of the trees in the plot (considering overall competition and relative vigour) and taking into consideration the time effect. These models constitute the ‘baseline’ to calculate impact, i.e. the difference in mortality and growth between the post-invasion and pre-invasion inventories. Our method is inspired by the one from Manion and Griffin’s^14^. First, it explicitly models mortality based on biological processes such as inter and intra-specific competition with other trees. Second, it allows testing whether mortality shifts are also observed on other non-host species, information which can be used to discriminate mortality due to a new pathogen from that caused by unspecific factors such as drought, frost or windstorms. Third, it explicitly includes growth losses as a pre-condition for mortality^26^. Growth losses are a common symptom of disease; the effect on growth is not only the result of impaired tree physiology, but also influenced by resource investment in tree defence or tissue repair instead of growth^27,28^. Growth is also a measure for vigour, and therefore growth losses can reveal whether a species attacked by a new pathogen is becoming less competitive over the other tree species. Combining growth and mortality may be used to observe inter- and intra-specific responses to mortality shifts. So, if a higher-than-normal mortality is coupled with a higher-than-normal growth by the surviving individuals of the same species, the turnover will increase but individual losses may be compensated. If increased mortality is coupled with a lower growth, the new pathogen may trigger niche replacement. We expect that the approach and the resulting models can be directly applied in forest management and planning.

## Results

**Figure 1:**
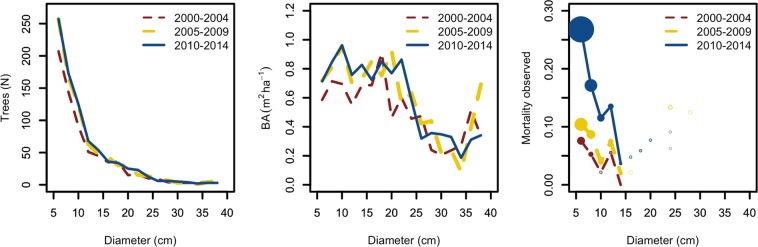
Number of ash trees observed in the Norwegian National Forest Inventory. Left: Trees by diameter class at intervals of 5 cm for the three periods studied (2000–04, 2005–09, 2010–14, corresponding to the 8^th^, 9^th^ and 10^th^ national forest inventories, respectively). Middle: basal area observed for the same periods. Right: Tree mortality observed for the same periods. In this case, the diameter classes were defined each 2 cm and the size of the points represents the number of trees dead (max N = 71). Lines do not link values when there are less than three trees dead.

The analysis of the observed frequencies of trees by diameter classes collected in three inventory datasets covering the years 2000–14 (Fig. 1) showed a trend of higher ash mortality, especially for small diameter trees. Particularly, records from the 10^th^ inventory (2010–2014) showed larger mortality rates (over two times) in small diameters compared to those observed before the first ash dieback records. In general, a 5-cm-diameter ash tree had a much larger probability to die in the post-invasion inventory than in earlier ones. The mortality during the 9^th^ inventory (2005–2009) was also slightly higher than the mortality observed during the 8^th^ inventory (2000–2004), but the differences were much smaller.

The impact of ash dieback on mortality rates was assessed by comparing mortality predictions based on pre-invasion inventories and later observations. Mortality was modelled by including basal area of the plot (BA_plot_) and tree diameter at breast height (dbh) (Table 1). In the case of the growth model, the model included the diameter at breast height and the basal area of the largest trees (the sum of the basal area of the trees larger than the subject tree) (BAL). The structure showed a good fit when including the plot effects, with a high coefficient of determination (R^2^ = 0.58). When plot information could not be retrieved, and the model was used to predict the growth of a tree at random, the predictive power was lower (R^2^ = 0.135). All variables included were significant (p-value < 0.001).

**Table 1:** Estimates for the mortality and growth models (Eqs. 1 and 2) and standard errors (in parenthesis).

Variables	Mortality model (Eq. 1)	Growth model (Eq. 2)
β_0_	−3.163 (0.404)	0.882 (0.226)
BA_plot_	−0.050 (0.017)	
BA_plot_ dbh^−1^	0.669 (0.078)	
dbh		0.718 (0.129) × 10
dbh^2^		−1.010 (0.265) × 10^3^
BAL		−0.208 (0.053) × 10
${\sigma }_{nfi}^{2}$	0.117	0.019
${\sigma }_{plot/NFI}^{2}$	1.528	0.536

BAplot: Basal area of all the species in the plot (m2 ha-1), dbh: ash tree diameter at breast height (cm), BAL: Basal area of larger trees (sum of the basal area of the trees larger than the subject tree) (m2 ha-1).

**Figure 2:**
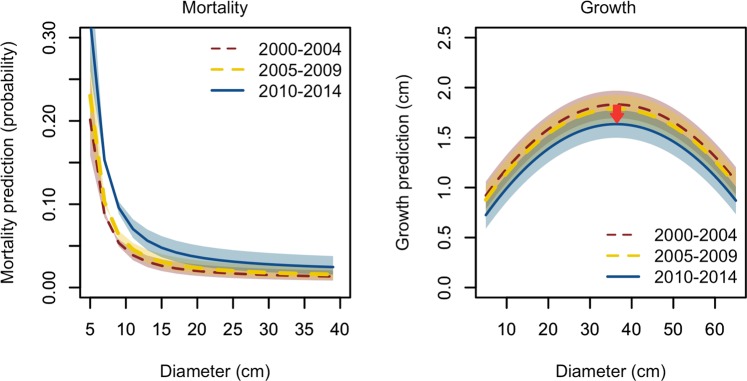
Modelled probability of ash tree mortality and growth. Mortality (left) and growth (right) predictions presented according to the tree diameter and for the three periods studied (2000–04, 2005–09, 2010–14, corresponding to the 8^th^, 9^th^ and 10^th^ national forest inventories, respectively). The earliest detection of ash dieback in Norway corresponds to the period (2005–09) and the disease is supposed to have spread along the west coast of the country in the latest period (2010–14). The models show the predictions for the median basal area (BA) on the mortality predictions and median basal area of larger trees (BAL) for the growth predictions. The shadowed areas represent the thresholds for the first and third quartile (BA = 19–31 m^2^ ha^−1^ and BAL = 12–25.2 m^2^ ha^−1^ respectively). The arrow illustrates the average growth reduction along inventories.

Changes along time in the mortality, expressed in between-inventory probability (${\sigma }_{NFI}^{2}$ = 0.117) and growth rates, expressed in cm of between-inventory diameter increment (${\sigma }_{NFI}^{2}$ = 0.019), were confirmed by the models proposed (Fig. 2). In fact, all the models showed the highest mortality in the last inventory period (µ_2000–2004_ = −0.24, µ_2005–2009_ =−0.11, µ_2010–2014_ = 0.35), as well as a reduction of growth (µ_2000–2004_ = 0.086, µ_2005–2009_ = 0.040, µ_2010–2014_ = −0.126) across inventories. On average, mortality was 74% higher and growth was 13% lower along the studied period for median values of basal area and basal area of the largest trees. A preliminary version of the models with dummy variables for each inventory was tested (instead of random factors), which confirmed the trends observed (p-value < 0.05).

**Figure 3:**
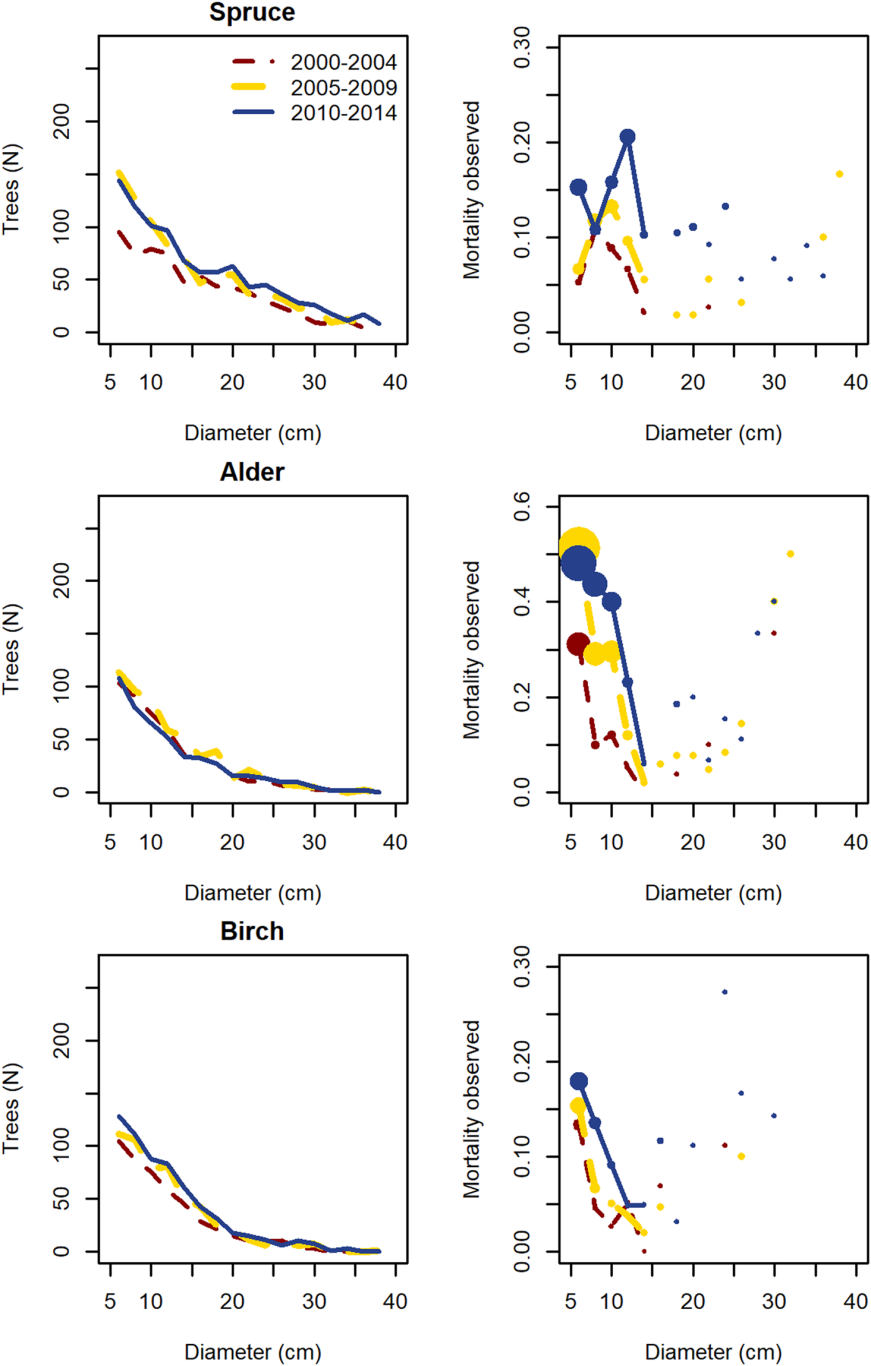
Diameter distribution and observed mortality of other species located in the ash plots. The distributions are presented for the three periods studied (2000–04, 2005–09, 2010–14, corresponding to the 8^th^, 9^th^ and 10^th^ national forest inventories, respectively) for Spruce, Alder and Birch.

Other tree species growing in the same plots were also explored. Among these, Norway spruce (*Picea abies*) was the most common tree, followed by birch (*Betula pendula*) and alder (*Alnus glutinosa*) (N = 1162, N = 697 and N = 689 trees, respectively). Contrary to ash, there was no consistent variation in mortality over time (Fig. 3). The species were also analysed with similar mortality and growth models (see Supplementary Table S1). For mortality, spruce and birch did not show any increment along inventories, whereas for alder, the model showed lower mortality in the first inventory (µ_2000–2004_ = −0.39, µ_2005–2009_ = 0.18, µ_2010–2014_ = 0.21). Concerning growth changes, in the case of spruce and birch these were negligible and for alder they did not show a clear pattern.

**Figure 4:**
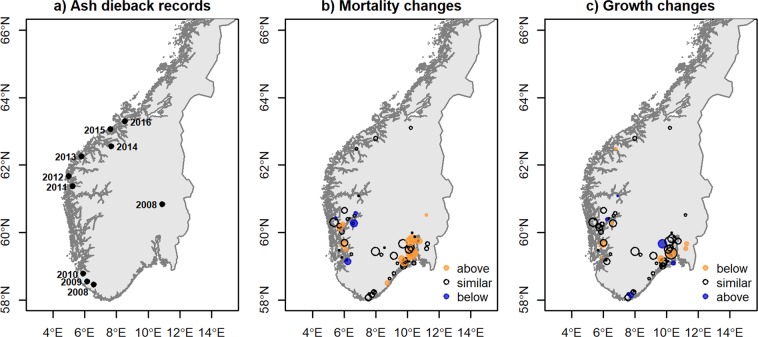
Changes in estimated plot mortality and growth. (**A**) Illustrates the first year of identification of the disease as a reference, the data is from ash dieback records from targeted inventories^24^. (**B**) Shows the mortality changes in a plot from the estimates of the random factors for the period 2010–14 compared to the period 2000–04 (Eq. 1) and (**C**) Shows the growth changes in a plot (Eq. 2). The values considered to be similar are within one standard deviation. The size of the circles represents the number of ash trees per plot. Maps generated with R (www.R-project.org); layer retrieved from ©Kartverket N250 (www.kartverket.no).

Mortality comparisons revealed regional patterns in terms of impact. The eastern areas of the country with longest disease history presented the largest increments in plot-related ash mortality (Fig. 4). In contrast, the west coast presented only four plots with higher-than-expected plot mortality, although the increase in ash mortality in these four plots was large. In this area, the disease started to spread around the 2009–2010^24^. In the northern areas, no changes in plot mortality were observed. The growth patterns showed similar regional distributions although the east-west difference was less pronounced.

**Figure 5:**
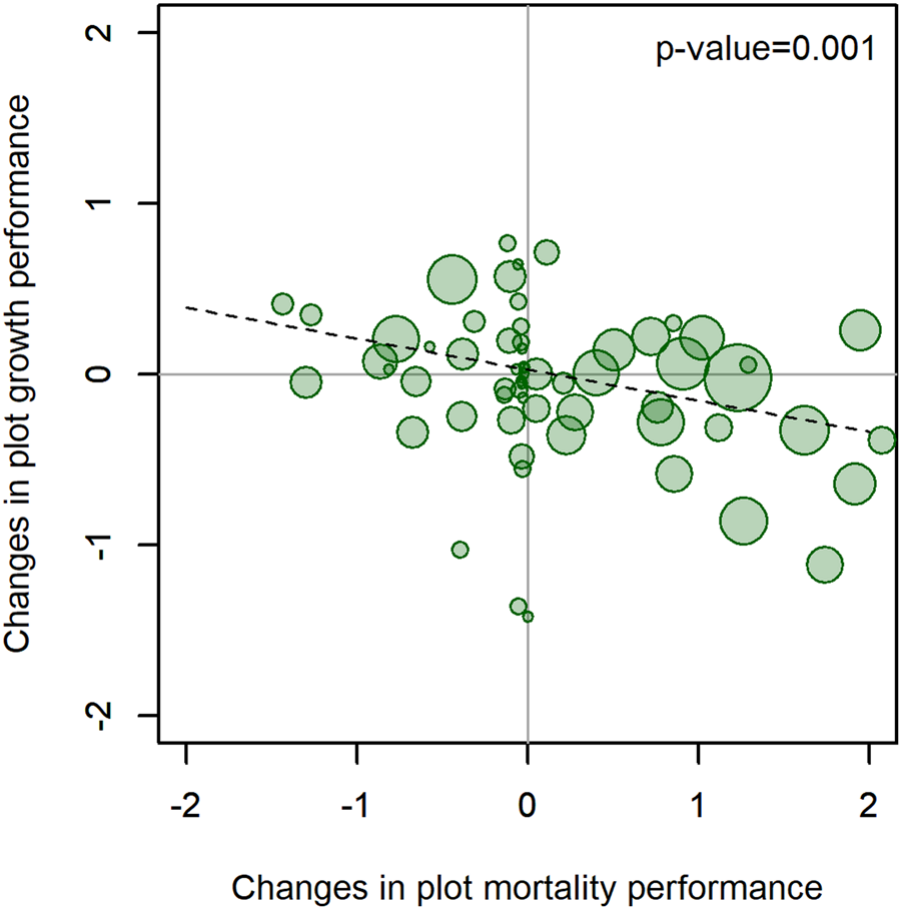
Plot level changes along time in ash tree mortality compared to growth performance. The changes are presented using the differences between the estimated plot random factors for mortality and growth in the period 2010–14 compared to the initial period 2000–04. The size of the circles represents the number of ash trees per plot.

We found no signs of growth compensation by persisting ash in areas with largest mortality (Fig. 5). In fact, plots with higher mortality tended to present a lower than expected growth rate by surviving ash whereas the other three species presented no trend (p-values 0.574, 0.660 and 0.317 for spruce, birch and alder, respectively, see Supplementary Figure S1). Besides the observed trends, both the number of ash trees as well as the number of plots with European ash increased from 720 trees in 102 plots from the 8^th^ inventory to 898 trees in 132 plots in the 10^th^ inventory (Table 2). In the 10^th^ inventory there were 33 new plots with ash that did not have ash in the 8^th^ inventory, with 108 trees growing on those plots. It must be noticed that some of the ash trees were removed as part of the management of those stands, ranging from 35 (9^th^ inventory) to 64 (10^th^ inventory). Around 50–65% of the trees removed presented less than 10 cm diameter; but since there were also more trees in this diameter class, the percentage was similar among the different diameter classes (ca 1% to 4%). Less than 20 trees were removed with diameters above 20 cm, including all the inventories.

**Table 2:** Descriptors of the main variables considered for analysis. dbh: diameter at breast height, BA: basal area. sd: standard deviation.

Variables		2000–04	2005–09	2010–14	2000–14
dbh_alive_ (cm)	*mean*	11.49	11.44	11.52	11.48
	*sd*	7.63	7.86	7.9	7.81
	*range*	5–69.4	5–74.6	5–74.6	5–74.6
dbh_dead_ (cm)	*mean*	8.9	12.15	14.63	12.22
	*sd*	7.27	7.03	10.48	8.82
	*range*	5–43.6	5–33.3	5–60	5–60
Growth (cm)	*mean*	1.19	1.09	0.94	1.09
	*sd*	1.13	0.93	0.86	1
	*range*	0–7.2	0–5.7	0–5.2	0–7.2
N_Ash trees_		720	875	898	2493
N_Ash trees dead_		32	58	136	226
N_plots with ash_		102	126	132	138
N_Ash trees removed_		38	35	64	137
BA_plot_ (m^2^ ha^−1^)	*mean*	25	24	26	25
	*sd*	9	10	10	10
Percentage of ash (%)	*mean*	38	40	35	38
	*sd*	28	30	26	28

## Discussion

An accurate quantification of the impacts caused by invasive organisms forms the basis for management strategies^29^. For fungal forest pathogens, tree mortality is the most commonly used variable to quantify losses. Since tree mortality is an inherent process of forest dynamics, defining a baseline against which mortality caused by a new pathogen can be compared presents a major challenge^30^. Manion and Griffin^14^ proposed a framework in which baseline mortality was extracted from tree inventory data collected across large areas. Because such an approach requires a lot of data, estimating mortality for minor tree species can be difficult. In this study, we developed a novel approach for minor tree species such as European ash (ca. 1% of trees in the NFI) in which mortality was explicitly modelled based on tree competition. We used pre- and post-invasion national forest inventories to identify shifts in terms of mortality and growth in ash, parallel to the spread of ash dieback. We found that in areas where ash dieback was more prevalent, plots tended to have a higher mortality than that predicted by the pre-invasion inventory - overall ash dieback increased mortality by 74% and mortality more than doubled in the smallest diameters. In areas not yet invaded, mortality mismatches amongst inventories occurred stochastically. The same pattern was found for growth, i.e. after the invasion ash had been growing 13% less than predicted by pre-invasion observations. By using the same comparative approach on other tree species co-occurring with ash in the same plots, we could exclude that mortality and growth differences in ash were due to unspecific abiotic factors, such as drought, spring frost, snow or windstorms. A higher-than-normal mortality could have been compensated by an increase of growth amongst surviving ash individuals. We observed the contrary. Higher-than-normal mortality correlated with a smaller-than-normal growth of the surviving ash trees, indicating that ash could be eventually replaced in the mid-term.

We shall also acknowledge some limitations in our approach. Firstly, the power of our analysis was limited by the low occurrence of ash trees in the plots available from the NFI. The Norwegian NFI has a grid of 3 × 3 km, and forest tree species with strong spatial structure such as ash are rarely captured as pure stands. Indeed, ash was very often recorded growing in mixed stands with other species, particularly with spruce. These conditions may not represent what happens in pure ash stands, or in mixed broadleaved stands in which disease development, and intra- and interspecific competition may be different. Coker^12^ estimated ash mortality rates by analysing targeted surveys reporting the proportion of trees that have died in sites across Europe and found mortality values as high as 85% in some pure European ash trials/plantations. Similarly, mortality values higher than 60% have been found in forests in Latvia and Lithuania^31,32^. The lower mortality recorded in the present study reflects probably country-specific differences in disease history: while the south-eastern Norwegian stands with the longest disease history were infected around 2006^24^, the data from targeted studies with the highest mortality in Central Europe come from forest stands with a disease history of 15 to 19 years^12^. In addition, the level of pathogen infection pressure can be expected to correlate positively with ash stocking density and thus differ between ash dominant stands typically included in targeted studies and stands where ash is a minor species, such as most of the plots now examined. Mismatches between observations in diseased areas and NFI data have been reported also previously. For instance, work on *Cryphonectria parasitica* and sweet chestnut in Spain showed contrasting results when comparing high incidence in managed stands with NFI data; the results indicated that instead of declining, chestnut population was increasing in volume, likely due to management abandonment of coppice stands^33^.

Additionally, the records showed that some ash trees had been removed from the stands, although there was no information concerning the purpose of the removals and whether they were part of a management plan. Possible explanations were that managers opted to cut valuable timber before the disease led to deterioration of wood quality, or, alternatively, opted to remove the more damaged trees during thinning, while maintaining the healthy and potentially tolerant ash individuals^34^ (although, arguably, ash is not particularly managed in Norway). In the first case, this could add to the model biases, resulting in an overestimation of the mortality. In the latter, this would mean that we have underestimated ash mortality. Since there is no information about the state of the trees prior to the removals, it is not possible to effectively evaluate their consequences in the models. However, the removed trees represented a similar percentage for each diameter class and accounted for less than 1% of the total recorded ash trees, which reduces the potential bias due to the removals.

We believe that by using NFI data we can obtain a broad and largely unbiased picture that can put in perspective observations from targeted surveys. NFI are based on a standardized sampling and measurements which allow an easy comparison across large areas, compensating the effect of local conditions disturbing the analyses. Furthermore, NFI data allowed us to determine a baseline mortality for comparative purposes. Our results are consistent with observations obtained from targeted monitoring of ash dieback in Norway^35^; these results showed similar patterns of mortality to those obtained from NFI, with the highest mortality rate for both the smallest trees and plots in the region with the longest disease history, in the south-eastern part of the country. On the other hand, the increased number of plots with ash and ash trees between the first and the last observations, despite the mortality, deserve also attention. A potential range expansion of ash due to climate warming, land use changes or increased numbers of surviving natural regeneration^2,12^ may be operating in parallel to the spread of ash dieback across Norway. Further forest inventories will be needed in future to monitor how ash stands develop in the long term after the infection.

Another limitation of our data was the time lag between inventories. We found a clear trend of increasing mortality in ash, particularly reflected in the last available NFI (2010–2014). A parallel analysis of ash growth showed also a reduction of radial increment, and a weak although significant negative correlation between both mortality and growth at plot level. A plausible explanation is that ash dieback is more likely to cause tree death in smaller trees than in large trees. This has implications when fitting our models, since growth losses in smaller trees may have been masked by death, as the 5-year-period between inventories was possibly too long for those trees to survive. When comparing pre- and post-invasion inventories, growth data was not accessible for small trees because in the next inventory the trees were dead. Regional differences in tree growth may also confound those caused by a new pathogen should the pathogen spread route parallel natural gradients. In our case, the fact that ash stands in the north were possibly growing less than ash stands in the south could have affected our capacity to detect an impact. However, regional growth differences should in theory be constant across inventories, and in our case, we only observed them in the last inventory.

Baseline mortality was modelled emulating the biological process underlying tree selection in natural forests. The structure of the data allowed the use of mixed models at tree level both for the mortality and the growth analyses in pure or mixed stands. In the case of the mortality model, the variables considered and model structure was based on the model of Sterba and Monserud^36^ for central Europe and that of Bollandsås^37^ for Norway. Mortality can be modelled based on tree characteristics with or without including the context of the plot. We tried to fit both, and the predictions were more accurate when plot variables were included. The probability of dying for a tree was modelled by considering the diameter of the tree, the basal area of the plot and the underlying biological processes: (i) larger trees within a plot are the ones showing the highest vigour and a (ii) higher BA increases competition for light and water amongst trees in the plot. Without including plot variables, the models underestimated mortality, particularly for young trees.

The negative correlation between mortality and growth indicates a lack of compensation of mortality by surviving ash trees within diseased plots. Although other reasons could explain this pattern, the most plausible one is that trees in the same plot are also diseased and therefore not able to make use of the resources made available through the decline of conspecifics. This is especially worrisome considering that ash is mostly represented in mixed stands and suggests that without intraspecific resilience it may be losing its niche. Another possibility would be that mortality removed too many trees in too short a time, so that the remaining trees, even though they were liberated from competition, did not have time to respond and fill the gap caused by the disease. Further follow up of these plots should confirm whether the observed pattern persists over time or represents a transitory situation. Finally, the observed appearance of ash in new plots across the inventories demonstrates the high dispersal capacity of the tree species^38^.

In our study, we have shown that NFI data can be used as a baseline to quantify impacts of a novel pathogen. Although other sources of mortality cannot be ruled out, due to the limitations of NFI data, our method represents a step forward from previous approaches, as mortality is explicitly modelled on a biological basis, and demonstrates that impacts on minor tree species growing in mixtures can be quantified. Including tree growth in our analysis allowed testing the resilience of the forest to possible pathogenic invaders and to devise the consequences in the mid-term. Concerning ash in Norway, we have shown that there has been an increasing mortality and reduction in growth after ash dieback was observed in the country. Although ash is a highly prolific species with a relatively high growth rate, it may not be able to buffer ash dieback, as no sign of growth compensation by the surviving trees was observed.

## Material and Methods

### Data sources

The tree level data were obtained from the Norwegian National Forest Inventory (NFI) entailing the period 1995–2014, corresponding to the 7^th^, 8^th^, 9^th^ and 10^th^ inventories (Table 2). The Norwegian NFI is a systematic inventory with permanent plots of 250 m^2^ in a 3 × 3 km grid, which are assessed on a five-year rotation basis. In this study, we considered all plots with ash trees (unique plots, N = 138) (Table 2). The NFI includes records concerning damage and tree mortality at plot and tree level, although it does not include the causes. We considered as living trees those trees that were categorized according to the NFI protocols as “the whole tree was alive”, otherwise they were considered dead (e.g. standing or lying dead). Those trees that were removed or not found were not considered. Trees were also excluded if they were categorized as dead in the previous inventory or there was no information on their status. All the ash trees assessed during the 7^th^ NFI (1995–99) were alive, therefore these observations were used as a tree status reference and not included as observations for the models. For alive trees, diameter increment in five years was calculated by considering the difference in diameter size between two subsequent inventories. Measurements resulting in negative growth increment were assumed to have measurement errors, which affected to 2.8% of the total available trees. The models were fit both including and excluding these records; the results did not show any large deviations in the model parameters. Therefore, it was decided to exclude records with negative growth from the final version of the models.

### Statistical analysis

#### Mortality models

The mortality models predict the probability of an ash tree to die in a 5-year period. The response variable is then the ash tree status, dead or alive. This type of analysis is often addressed by using a logistic function. In our case, there is an additional feature: since the records are based on forest inventory data, where trees are grouped in plots, and measured in three consecutive inventories, the data forms a three-level hierarchy (i.e. a tree *i* is alive or dead in plot *j*, in time *t*). To accommodate this, we took a generalized linear mixed model approach, where the data can be grouped by using random factors. Thus, the general structure was modelled after a binary logistic and using the logit equation as link function, where the distribution of the binomial response variable ${Y}_{ijt}$ for a tree *i* in plot *j* at time *t* depends on a set of $k$ explanatory variables ${{\boldsymbol{X}}}_{ijt}=({X}_{ijt1},{X}_{ijt2},\ldots {X}_{ijtk})$, following:

$${Y}_{ijt}={\rm{dead}}(1)\,{\rm{or}}\,{\rm{alive}}(0)$$

$${Y}_{ijt} \sim {\rm{binomial}}(1,{P}_{ijt})$$

where the link function is:

1 $$g(P)=logit(P)=log\left(\frac{P}{1-P}\right)={\beta }_{0}+{\beta }_{1}{X}_{1}+{\beta }_{2}{X}_{2}+\ldots +{\mu }_{t}+{\mu }_{jt}$$

where ${P}_{ijt}$ is the probability of a tree mortality *i* in plot *j* at time *t*, ${\boldsymbol{\beta }}=({\beta }_{0},{\beta }_{1},\ldots {\beta }_{n})$ are the model parameters to be estimated, and *μ*_*t*_ *and μ*_*jt*_ are the deviations from the intercept of amount *μ* for plot *j* in the inventory *t* (as between-plot and between-inventories random factor), considered independent and identically distributed with mean = 0 and a constant variance ${\sigma }_{plot}^{2}$ and ${\sigma }_{nfi}^{2}$. Although initially we contemplated the option of including random factors also affecting the slope of the variables, this resulted in difficulties to fit the models, given the limited amount of data.

From this general structure, we fitted several models aiming at describing the tree size and the competition (largely following the variables proposed by Monserud and Sterba^36^) by considering several predictive variables both at tree and plot level. To include these variables in the final version of the model, they had to be significant at the 0.05 level, contributed to the predictive power of the model, and resulted in parsimonious models that would facilitate interpretation. For the model selection, the performance was assessed based on their AIC and BICs estimates.

#### Growth models

The growth models predicted the diameter increment of a tree during the 5-year-period between two subsequent NFI measurements, expressed as ${I}_{5ij}$. In this case we also accommodated the two-level hierarchy by using a linear mixed model approach, where the data was grouped at plot level by using random factors. The model depended on a set of $k$ explanatory variables ${{\boldsymbol{X}}}_{ij}=({X}_{ij1},{X}_{ij2},\ldots {X}_{ijk})$, following:

2 $${I}_{5ijt}={\beta }_{0}+{\beta }_{1}{X}_{1}+{\beta }_{2}{X}_{2}+\ldots +{\mu }_{t}+{\mu }_{jt}+{\mu }_{ijt}$$

where ${I}_{5ijt}$ is the diameter increment of a tree *I* in plot *j* at time *t*, ${\boldsymbol{\beta }}=({\beta }_{0},{\beta }_{1},\ldots ,{\beta }_{n})$ are the model parameters to be estimated, and *μ*_*t*_, *μ*_*jt*_ *and μ*_*jit*_ are the deviations from the intercept of amount *μ* for tree *i* in plot *j* in the inventory *t* (as between-trees, between-plot and between-inventories random factors, respectively), considered independent and identically distributed with mean = 0 and constant variance ε, ${\sigma }_{plot}^{2}$ and ${\sigma }_{nfi}^{2}$.

In addition, the same model structures for mortality and growth were applied to the three most abundant species after ash in the same plots (*Picea abies*, *Betula spp*. and *Alnus spp*.), in order to test whether mortality and growth differences between inventories were specific to ash or due to phenomena affecting all the other tree species. Finally, the resulting plot estimates of the random factors from the growth and mortality models were visually inspected to explore patterns that would indicate geographical differences in mortality or growth. The model parameters were fitted based on restricted maximum likelihood by using the packages *lme4*^39^ (Eq. 1, mortality models) and *nlme*^40^ (Eq. 2, growth models), since the former can handle generalized models (needed for the mortality models), while the latter makes use of the Gaussian link function and, for us, shows and handles better the covariance structure and degrees of freedom. The maps were prepared with the package *rgdal*^41^ of the R statistical software^42^.

## Supplementary information

Supplementary information.

## Data Availability

The authors confirm that all data underlying the findings of this study are fully available from The Norwegian Institute of Bioeconomy Research, partly openly accessible at https://landsskog.nibio.no/ and https://ssb.no/en/jord-skogjakt-og-fiskeri/statistikker/lst/aar?fane=arkiv#content and partly by contacting the institute through Aksel Granhus (aksel.granhus@nibio.no). The data used in this study are third-party data. Data are collected as part of a national monitoring program. The use of data is restricted by the data use policies of the national monitoring program of which they are part. Data are available upon request and in accordance with the guidelines of the monitoring program of which they are part.
