# Supplementary material for: The invasive forest pathogen Hymenoscyphus fraxineus boosts mortality and triggers niche replacement of European ash (Fraxinus excelsior)
Source: Sci Rep. 2020 Mar 24;10:5310. doi: 10.1038/s41598-020-61990-4 (PMC7093550; doi:10.1038/s41598-020-61990-4)
Supplement: Supplementary file 1 — Supplementary information. [file 41598_2020_61990_MOESM1_ESM.docx]

**Supplementary Information**

**Article in Scientific Reports**

**The invasive forest pathogen Hymenoscyphus fraxineus boosts mortality and triggers niche replacement of European ash (Fraxinus excelsior)**

Olalla DÍAZ-YÁÑEZ, Blas MOLA-YUDEGO*, Volkmar TIMMERMANN, Mari Mette TOLLEFSRUD, Ari M. HIETALA, Jonàs OLIVA

(* contact person: blas.mola@uef.fi)

**Table S1. Estimates for the mortality and growth models (equation 1 and 2) and standard errors (in parenthesis) on plots where ash is present.** BA_plot_: Basal area of all the species in the plot (m^2^ ha^-1^), dbh: ash tree diameter at breast height (cm), BAL: Basal area of larger trees (sum of the basal area of the trees larger than the subject tree) (m^2^ ha^-1^). All variables were significant at the 0.05 threshold.

Mortality model (Equation 1)

| Variables | Ash | Spruce | Birch | Alder |
| --- | --- | --- | --- | --- |
| β_0_ | -3.163 (0.404) | -6.268 (0.465) | -4.303 (0.343) | -2.398 (0.421) |
| BA_plot_ | -0.050 (0.017) |  |  | -0.113 (0.016) |
| BA_plot_ dbh^-1^ | 0.669 (0.078) |  |  |  |
| dbh^-1^ |  | 14.612 (2.687) | 7.791 (2.490) |  |
| dbh^2^ (x10^-3^) |  | 2.175 (0.371) |  | 4.574 (0.637) |
| BAL (x10) |  | 0.693 (0.110) | 0.557 (0.128) |  |
| BAL ln(dbh)^-1^ |  |  |  | 0.355 (0.028) |
| µ_2000-2004_ | -0.242 | -0.044 | -0.015 | -0.392 |
| µ_2005-2009_ | -0.108 | 0.022 | -0.002 | 0.180 |
| µ_2010-2014_ | 0.350 | 0.022 | 0.016 | 0.212 |
|  |  |  |  |  |
| σ^2^_NFI_ | 0.117 | 0.010 | 0.003 | 0.114 |
| σ^2^_plot/NFI_ | 1.528 | 1.826 | 0.820 | 0.523 |

Growth model (Equation 2)

| Variables | Ash | Spruce | Birch | Alder |
| --- | --- | --- | --- | --- |
| β_0_ | 0.882 (0.226) | 2.401 (0.266) | 0.842 | 0.246 |
| BA_plot_ |  |  |  |  |
| BA_plot_ dbh^-1^ |  |  |  |  |
| dbh (x10) | 0.718 (0.129) | 0.63 (0.17) | 0.28 (0.056) | 1.111 (0.234) |
| dbh^2^ (x10^-3^) | -1.010 (0.265) | -0.789 (0.360) |  | -2.651 (0.671) |
| BAL (x10) | -0.208 (0.053) | -0.563 (0.048) | -0.215 (0.046) | -0.172 (0.046) |
| BAL ln(dbh)^-1^ |  |  |  |  |
| µ_2000-2004_ | 0.086 | <0.001 | 0.046 | 0.086 |
| µ_2005-2009_ | 0.040 | <0.001 | 0.005 | -0.067 |
| µ_2010-2014_ | -0.126 | <0.001 | -0.051 | -0.020 |
| σ^2^_NFI_ | 0.019 | <0.001 | 0.005 | 0.012 |
| σ^2^_plot/NFI_ | 0.536 | 0.567 | 0.127 | 0.164 |
| σ^2^_e_ | 0.494 | 0.930 | 0.311 | 0.398 |


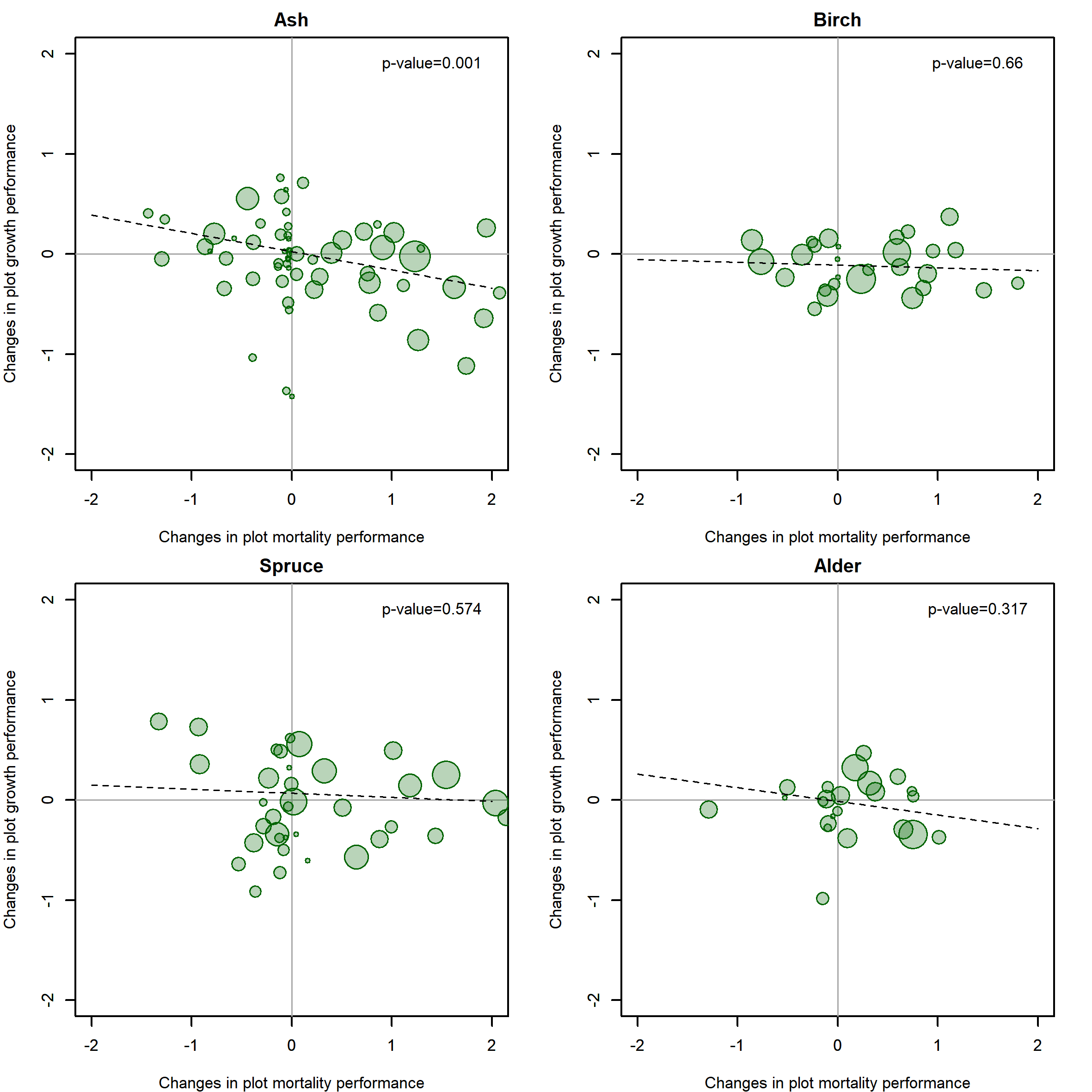


**Figure S1. Plot level changes along time in ash tree mortality compared to growth performance.** The changes are presented using the differences between the estimated plot random factors for mortality and growth in the period 2010-14 compared to the initial period 2000-04 on plots where ash is present. The size of the circles represents the number of trees of that species per plot, and the p-values relate to the significance of the weighted trend. The corresponding unweighted p-values are 0.031, 0.85, 0.85 and 0.94 for ash, spruce, birch and alder, respectively.
